# Feeding Behaviour in Group-Housed Growing-Finishing Pigs and Its Relationship with Growth and Feed Efficiency

**DOI:** 10.3390/vetsci12020168

**Published:** 2025-02-13

**Authors:** Miriam Piles, Llibertat Tusell, Mónica Mora, Carolina Garcia-Baccino, Denis Cudrey, Claire Hassenfratz, Marie-José Mercat, Ingrid David

**Affiliations:** 1 Institute of Agrifood Research and Technology (IRTA)—Animal Breeding and Genetics, Caldes de Montbui, 08140 Barcelona, Spain; llibertat.tusell@irta.cat (L.T.); monica.mora@irta.es (M.M.); 2 NUCLEUS, 35650 Le Rheu, France; c.garciabaccino@nucleus-sa.com; 3 Alliance R&D, 35740 Pace, France; dcudrey@axiom-genetics.com (D.C.); claire.hassenfratz@ifip.asso.fr (C.H.); marie-jose.mercat@ifip.asso.fr (M.-J.M.); 4 AXIOM, 37310 Azay sur Indre, France; 5 IFIP—Institut du Porc, 35740 Pacé, France; 6 GenPhySE, French National Research Institute for Agriculture (INRAE), National Veterinary School of Toulouse (ENVT), Université de Toulouse, 31326 Castanet Tolosan, France; ingrid.david@inrae.fr

**Keywords:** feeding behaviour, social ranking, dominant/subordinate, feed efficiency, pigs

## Abstract

Feed consumption and feeding patterns influence the individual feed efficiency in group-housed livestock species. Using the meal as the unit of feeding behaviour, the main objectives of this research were to identify feeding behaviour (**FB**) traits that may indicate an individual’s rank within the social hierarchy or its level of dominance among pen mates and to assess the relationship between growth and feed efficiency with the identified traits, as well as those describing individual feed consumption patterns. Data from 5516 pigs during the fattening period were used. Pens were equipped with an automatic concentrate feeder that recorded individual feed intake, time spent at the feeder, and body weight at each visit. A meal criterion was established. Then, different FB traits were computed: number of meals, number of visits to complete a meal, occupation time to complete a meal, time between first and last visit within a meal, feeding rate, feeding rate to complete a meal, and interval between meals. Social ranking (**SR**) traits were also calculated: position/order in which each animal accessed the feeder, ratio of visits to the feeder during preferred times, distribution among the cage mates of the total daily feed, number of visits, number of meals, and occupation time. Pigs that eat more and faster tend to have a poorer feed efficiency and higher final weight. Animals that eat more, more times, and occupy the feeder longer, eat mainly in the preferred period. They could be considered dominant, while others have to adapt their feeding schedules to off-peak times.

## Simple Summary

Feed consumption and feeding behaviour play a key role in the feed efficiency of livestock housed in groups. This study examined feeding behaviour traits, social hierarchy indicators, and their relationships with growth and feed efficiency in 5516 pigs during the fattening period. Using data from automated feeders, we identified traits such as meal frequency, feeding rate, and feeder occupation time, among others. Results showed that pigs which ate more and faster had poorer feed efficiency and higher final weights. Dominant pigs tended to eat during preferred feeding times, leaving others to adapt to less favourable schedules. Understanding these patterns can help optimize group management and feeding strategies.

## 1. Introduction

Improving the feed efficiency (**FE**) of pigs is a priority area of research [1] due to the high economic cost of feeding, which is estimated to be approximately 65% of the cost of producing a pig [2]. This improvement may be targeted directly or achieved indirectly through the selection of other genetically correlated traits like growth or body fatness [3]. Feed consumption and feeding patterns influence the individual FE in group-housed livestock species. These in turn can be influenced by the impact of group mates on the individual performance due to factors like competition for feeder access, aggression, and more [4,5,6]. Thus, the level of competition at the feeder has been linked to variations in production performance within pens [7] and alterations in feeding behaviour [8]. In group-housed animals, competition for feed resources is a common challenge [9]. This issue becomes particularly significant for animals that are unable to leave the group and must confront conflicts directly. Feeding typically occurs in specific areas and at fixed times, which intensifies competition and promotes defensive behaviours, often resulting in increased stress and aggression [10]. The intensity of competition depends on resource availability, being lower when feed is abundant and higher when it is limited or easily defended. However, even when animals have ad libitum access to feed, social and physiological factors—such as metabolic demands and hormonal rhythms that regulate feeding behaviour—can contribute to competitive interactions [11]. Social facilitation, where animals are stimulated to eat upon observing others feeding, can further exacerbate these conflicts [12].

Using electronic feeders in nucleus or experimental farms enables the collection of detailed data on feed intake and feeding patterns over a specified period. Additionally, the data collected by electronic feeders can be utilized more broadly to identify new phenotypes that help to understand the mechanisms by which an animal influences the performance of its group mates. Some of these traits could indicate an animal’s social ranking or dominance within the group. These traits might include the distribution of feed consumption or feeder occupation time among group mates, as well as the order in which animals access the feed trough during their visits to complete a meal or the time of day when they do it.

Many studies on feeding behaviour (**FB**) focus on traits derived from feeder visits, such as the number of visits, time spent at the feeder, feed intake, and feeding rate per visit, or on daily and hourly aggregates of these traits. However, the devices and methods used to record these data greatly influence the definition, biological relevance, quantity, and average duration of feeder visits [13]. As a result, findings from studies employing different techniques are often difficult to compare, with meaningful comparisons only possible when similar recording devices and methodologies are used to define FB traits. Given that feeder visits are influenced by random factors, some researchers suggest using meals as a more reliable unit for evaluating FB traits [13]. Meals are considered the smallest biologically meaningful unit of feeding behaviour, as they align with the biological concept of satiety [14].

Using the meal as the unit of FB, the objectives of this research are as follows: (i) to characterise the daily and hourly FB patterns of growing finishing pigs; (ii) to identify FB parameters/traits that may indicate an individual’s rank within the social hierarchy or its level of dominance among pen mates; and (iii) to assess the relationship between growth and feed efficiency with the identified traits, as well as those describing individual feed consumption patterns. This study considered social dynamics aspects, such as dominance and feeder access order, to explore how these interact with traits of economic interest in pig production. This information was expected to contribute to developing management and breeding strategies for pig farms by taking advantage of the automatic measurement of traits to identify the most efficient or dominant animals, eliminating the need for visual monitoring.

## 2. Material and Methods

### 2.1. Animals and Housing

Data were collected in compliance with the national regulations on livestock welfare in France.

For this study, data from 5516 pigs (1369 males from **breed 1**, 1192 females from **breed 2**, and 2955 females from **breed 3**) from the INRAE-France Génétique Porc facility (UEPR, INRAE, Rennes pig experimental unit, Le Rheu, France, 2018, https://ue-3p.isc.inrae.fr/) (accessed on 1 January 2025), collected between 2017 and 2019, were used. The pigs were raised during a fattening period of approximately 100 days (from around 63 to 162 days of age), with day 0 being incomplete, in pens, each equipped with a Genstar v3 Acemo^®^ automatic concentrate feeder and animal weighing scales. During each visit to the automatic concentrate feeder, individual feed intake (**FI**), time spent at the feeder, and body weight (**BW**) were recorded. The animals were divided into 52 batches and housed in 440 separate pens, with each pen containing only one breed. Animals were housed in pens of 14 pigs (ranging from 9 to 15) with 9 pens per room (of which at least 6 were used in the study), and the initial weight was similar for all breeds and pens. This density of animals per pen was determined based on legislative requirements for the minimum space per animal and to avoid exceeding 95% of the occupation time of the feeder. This was crucial to avoid potential impacts on the results of the research, given that excessive feeder occupation could influence both feeding behaviour and social dynamics.

### 2.2. Growth and Feed Efficiency Traits

The weight of each pig was automatically recorded at the feed station. From this information, the average daily gain was computed for each animal as the slope of the linear model of body weight on the animal’s age using the function lm() of the R Package “stats” [15]. This variable was named average daily gain regression (**ADGreg**). The feed conversion ratio (**FCR**, in units) was determined by dividing the average daily feed intake (**ADFI**) of the animal during the follow-up period by its ADGreg. Finally, the residual feed intake (**RFI**) was computed as the residual of the linear model of the feed intake on ADGreg, metabolic weight (**MW**), and lean percentage (**Lean%**) using the function lm() of the R Package “stats” [15]). The MW was calculated as ${\frac{\mathrm{BWs}+\mathrm{SW}}{2}}^{0.75}$. The terms BWs and SW represent the body weight at the start of the follow-up period (~23 kg) and the body weight at the end of the follow-up period (~120 Kg), respectively.

### 2.3. Data Screening and Definition of Meals

Cages containing dead or culled animals, along with incomplete records from the first and last days of the observation period, were excluded from the analysis. Additionally, data cleaning involved removing records with missing values or incorrect animal identifications, which accounted for less than 0.07%, 0.10%, and 0.09% of the dataset, for breed 1, breed 2, and breed 3, respectively.

The meal criterion was established following the methodology proposed by Howie et al. [14], as well as defined in [16]. This method analyses changes in the probability of animals starting to feed (**Pstart**) relative to the time since their last visit. It calculates the observed Pstart within each minute as the ratio of intervals starting between t and t + 1 min to intervals starting after t minutes. The meal criterion is determined where Pstart reaches its minimum, identified as the point where the change in Pstart shifts from negative to positive values. A rolling average over 5 min intervals is applied to smooth out random fluctuations in the data.

### 2.4. Feeding Behaviour Traits

All analysed FB traits are presented and described in detail in Table 1. The interval between meals (**IBM**, in s) was calculated as the difference between the start times of two consecutive meals.

### 2.5. Social Ranking Traits for an Animal Within the Cage Group

Social ranking (**SR**) traits were also calculated as potential indicators of an animal’s social ranking or dominance within the group. The position/order in which each animal accessed the feeder was recorded and calculated as described in Piles et al. [16]. This variable was standardised to account for the fact that the number of visits to the feeder is not constant in all meals or all cages. Based on this information, the mean standardized order of successive visits to complete each meal was calculated for each animal. This trait was referred to as **Position**. We also defined the variable **prefTime.a** as the ratio of visits to the feeder performed during the period from 8:00 to 20:00 h and **prefTime.b** as the ratio of visits performed during the period from 8:00 to 10:00 and from 15:00 to 17:00 h. In addition, for each feeder and each day within the observation period, the distribution among the cage mates of the total feed FI (**rateFI**), the number of visits (**rateNV**), the total number of meals (**rateNM**), and occupation time (**rateOT**) were calculated. This was performed by taking the ratio of each animal’s total values relative to the total values of all cage mates on that specific day.

### 2.6. Statistical Analysis

To eliminate anomalous records, data were processed separately for each breed. Outliers were removed using the robust Mahalanobis distance criterion based on the variables TM, FIM, and IBM. We selected this approach because it takes into account the correlation between variables, making it particularly suitable for identifying anomalous records in datasets with related variables, as was the case in this study. Additionally, this criterion is less sensitive to the influence of outliers compared to traditional approaches. We first calculated the robust distance of each observation $x_{i}$ to the mean of the multivariate Gaussian distribution of the data as follows:

$$rd\left(x_{i}\right)=\sqrt{\left(x_{i}-{µ}_{mcd}\right)\prime \;S_{mcd}^{-1}\;\left(x_{i}-{µ}_{mcd}\right)}$$

In this equation, ${µ}_{mcd}$ and $S_{mcd}^{-1}\;$ are the robust Minimum Covariance Determinant (MCD) estimates of the mean vector and covariance matrix, respectively, obtained from the 75 % subset of data with the smallest covariance determinant using the cov.mcd function from the MASS Package in R [15]. An observation was classified as an outlier if $rd\left(x_{i}\right)>5\times \sqrt{\chi_{p,0.999}^{2}}$, in which $\chi_{p,0.999}^{2}$ is the Chi-Square critical value at the 0.001 significance level with 3 degrees of freedom of the p variable. The percentage of outliers was 0.31% for breed 1, 0.28% for breed 2, and 0.44% for breed 3.

After removing outliers, daily values for each FB trait were obtained by summing the values for each day and animal. Hourly values were calculated by summing the values for each hour and animal and dividing by the number of days.

For each breed, Pearson correlations were separately calculated between individual averages of social ranking traits and individual averages of feeding behaviour, growth, and feed efficiency traits. This was accomplished using the “cor()” function from the “DescTools” library in R [18].

## 3. Results

### 3.1. Growth and Feed Efficiency Traits

Table 2 presents the descriptive statistics of growth and FE traits for the three different pig breeds. Values obtained are difficult to compare with values obtained in the literature due to different management practices and protocols inherent to these three breeds.

### 3.2. Intervals Between Visits and Meal Criterion Definition

**Figure 1:**
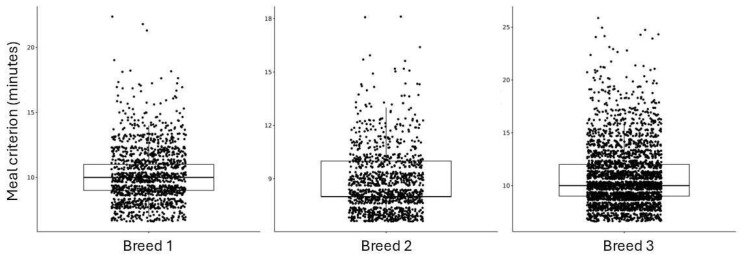
Boxplots of individual meal criterion for each animal from each breed.

Figure 1 shows the boxplots of the individual meal criterion, which was estimated according to Howie et al. [14] for each animal of each breed. There is a great variability in those values, which reached 25 min for some individuals, but the median is between 9 and 10 min.

### 3.3. Feeding Behaviour

**Figure 2:**
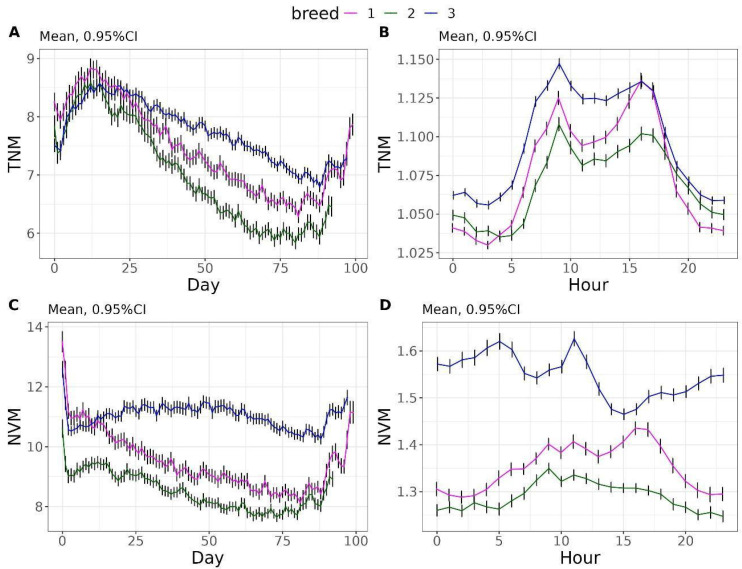
Daily evolution of total number of meals (TNM) and number of visits to the feeder to complete a meal (NVM) (**A**,**C**). Hourly evolution of TNM and NVM (**B**,**D**).

This section illustrates the evolution of feeding behaviour traits during the fattening period as well as their daily pattern. Figure 2 shows the evolution of TNM and NVM traits. The TNM reaches the peak around day 12–15 for the three breeds, with values of 8.83, 8.58, and 8.57 for breeds 1, 2, and 3, respectively. From this moment onwards, it gradually reduced to reach a minimum at around day 81–88, with values of 6.29, 5.84, and 6.79 for breeds 1, 2, and 3, respectively (Figure 2A). Hourly, the three breeds exhibited two peaks, occurring around 9:00 h and 17:00 h. During the first peak (around 9:00 h), the values were 1.13, 1.11, and 1.15 for breeds 1, 2, and 3, respectively. For the second peak (around 17:00 h), the values were 1.14, 1.10, and 1.14 for breeds 1, 2, and 3, respectively (Figure 2B). The daily pattern of NV is shown in Figure 2C. The TNV averaged 9.50, 8.50, and 11.03 for breeds 1, 2, and 3, respectively. Hourly, breed 2 showed the lowest TNV, reaching a maximum of 1.35 at hour 9, while breed 3 reached its maximum at hour 11 with a value of 1.62 (Figure 2D).

**Figure 3:**
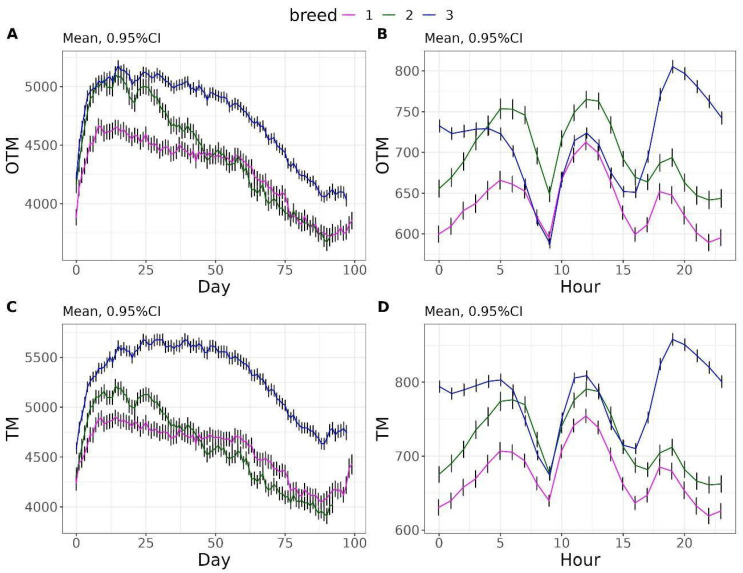
Daily evolution of occupation time in a meal (OTM) and time to complete a meal (TM) (**A**,**C**). Hourly evolution of OTM and TM (**B**,**D**).

The OTM reached its peak on day 8 in breed 1 with a value of 4662.66 s (77.71 min), on day 14 in breed 2 with a value of 5103.38 s (85.06 min), and on day 15 in breed 3 with a value of 5179.32 s (86.32 min). From these points onward, the OTM values steadily declined, reaching their minimum around day 90 with an average value for the three breeds of 3816.91 s (63.61 min) (Figure 3A). Similarly, TM reached its peak in the first third of the control period: on day 14 with a value of 4902 s (82 min) for breed 1, on day 14 with a value of 5213 s (87 min) for breed 2, and on day 24 with a value of 5681 s (95 min) for breed 3 (Figure 3C). These values decreased over the following days except for breed 3 for which a plateau was reached until day 39. In the hourly pattern and in the three breeds, two troughs can be observed in both OTM and TM around 9 h and 16 h (Figure 3B,D).

**Figure 4:**
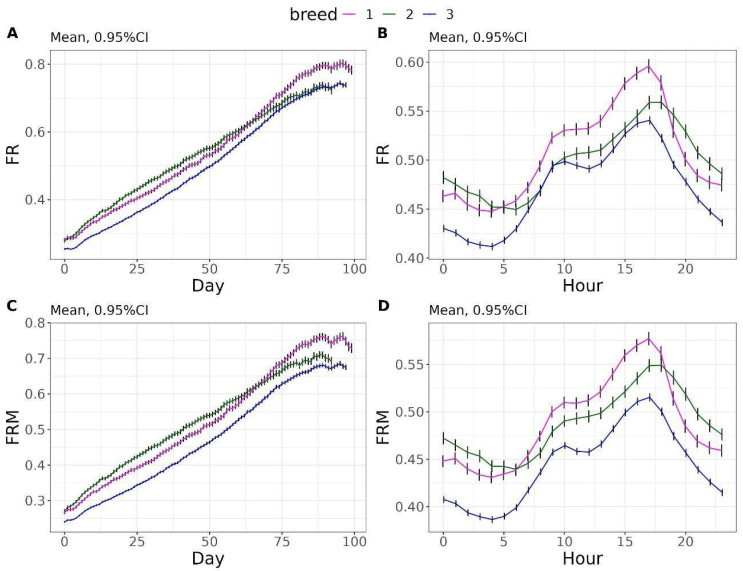
Daily evolution of feeding rate (FR) and feeding rate to complete a meal (FRM) (**A**,**C**). Hourly evolution of FR and FRM (**B**,**D**).

The FR and FRM exhibited a daily increasing pattern over the days, reaching a plateau value of 0.80 g/s by day 88 for breed 1 and 0.73 g/s by day 88 for breed 2 and 3 (Figure 4A,C). Similarly, the hourly patterns for FR and FRM were similar, with two peaks occurring around 10 h and 17 h, with the latter being the maximum peak for these traits. At this time, FR values were 0.59 g/s, 0.56 g/s, and 0.54 g/s for breeds 1, 2, and 3, respectively (Figure 4B), while FRM values were 0.57 g/s, 0.55 g/s, and 0.51 g/s for breeds 1, 2, and 3, respectively (Figure 4D).

**Figure 5:**
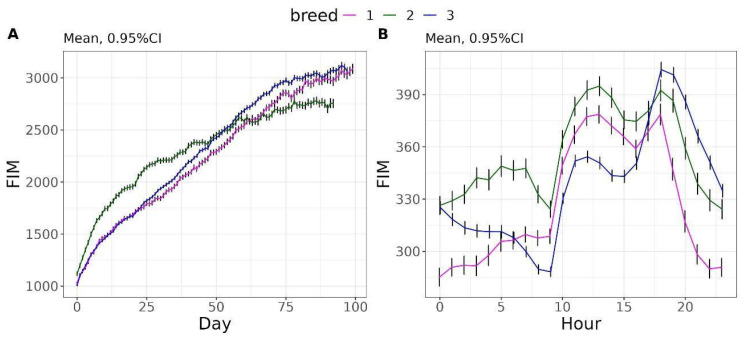
Daily evolution of feed intake (FIM) (**A**) and hourly evolution of FIM (**B**).

The FIM gradually increased over the days, reaching an average of 2967 g for breed 1, 2753.49 g for breed 2, and 3075.83 g for breed 3 by day 92, up from an average of 1022 g, 1118 g, and 1012 g on day 0 for breeds 1, 2, and 3, respectively (Figure 5A). Regarding the hourly pattern, all three breeds show a peak around 13:00 h and 18:00 h (Figure 5B).

### 3.4. Correlations Between Traits

**Figure 6:**
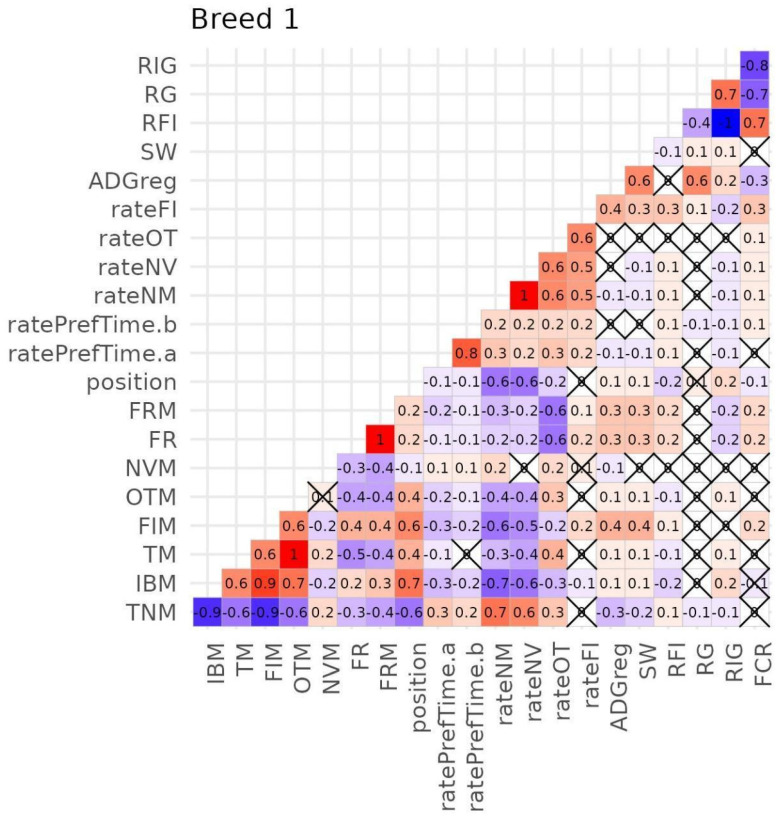
Phenotypic correlations between productive performance (i.e., growth and feed efficiency), feeding, and social behaviour traits in pigs from breed 1. Black crosses (X) indicate non-significant correlations between variables. The colour and its intensity correspond to the value of the correlations, ranging from high and positive (dark red) to high and negative (dark blue). Refer to Table 1 for the abbreviations and detailed descriptions of the variables.

The correlation patterns between the different traits were quite similar across the three breeds, even though no comparisons were made. Figure 6 displays the phenotypic correlations of pigs from breed 1, which are the only ones discussed. Results corresponding to breed 2 and 3 can be found in the Supplementary File S1. A *p*-value of <0.05 was used as the threshold to identify correlations as statistically significantly different from zero.

The correlation between SW and ADGreg was moderate (0.6). In general, the correlations between growth traits (SW and ADGreg) and feed efficiency traits (RFI, RG, RIG, FCR) were almost null, except for the correlation between FCR and ADGreg, which was low (−0.3), and the correlation between ADGreg and RG, which was moderate (0.6). However, it must be considered that SW was not measured at a fixed age but at the slaughtered time targeting a live weight of ~120 kg, and this can impact the estimated correlations with the other traits.

The phenotypic correlations with FB and SR traits were equal for SW and ADGreg and globally low or very low. The highest values were found for the correlations between growth traits and FIM and rateFI (0.4), followed by the correlation between growth traits and FRM, FR, and TNM being positive for the first two traits (0.3) and negative for the last one.

The phenotypic correlations among FE traits were all high or very high except the one between RFI and RG, which was negative and low (−0.4). However, those traits were not correlated with FB or SR traits, except RFI and FCR with rateFI, which showed a low and positive value (0.3).

For some pairs of FB traits, the correlation was high or very high: TNM and IBM (−0.9); FIM and TNM (−0.9); FIM and IBM (0.9); and OTM and TM (1.0). The correlations were moderate to high between TM and TNM (−0.6), TM and IBM (0.6), FIM and TM (0.6), OTM and TNM (−0.6), OTM and IBM (0.7), and OTM and FIM (0.6). FR and FRM showed low correlations with the other FB traits, ranging from 0.3 to 0.4, while NVM was not correlated with other FB traits except FR and FRM. The correlations between FB and SR traits ranged from null or very low to high values. The most remarkable is the moderate to high correlation between TNM, IBM, and FIM and the variables position, rateNM and rateNV, and between FR and FRM and rateOT. Thus, rateNM and rateNV were strongly positively correlated with TNM, at 0.7 and 0.6, respectively, while they were negatively correlated with IBM, at −0.7 and −0.6, respectively. Additionally, they showed moderate negative correlations with OTM and FIM, ranging from −0.4 to −0.6.

Regarding correlations within markers of hierarchical position or dominance among cage mates, rateNM and rateNV were completely correlated between them and moderately correlated with position (−0.6) and rateOT (0.6) and rateFI (0.5).

The variable ratePrefTime.a and ratePrefTime.b had a negligible negative correlation with position but both were slightly correlated with rateNM, rateNV, rateOT, and rateFI.

## 4. Discussion

### 4.1. Feeding Pattern

As the animal adapts to the new environmental conditions at the start of fattening, TNM, OTM, and TM increase until they reach a maximum around 14 days after the start of fattening. Younger pigs tend to consume feed in more frequent but smaller portions, while older pigs reduce the number of feeding events, taking in larger meals with a higher feeding rate. This result agrees with findings of previous studies [19,20]. This shift in feeding patterns may result from the increase in stomach size, which expands significantly as pigs grow—from just 30 mL at birth to around 3.5 L by the time they reach finishing weight [21]. This suggests that younger pigs, such as those weighing around 20 kg, may be limited by stomach capacity, leading them to take smaller and more frequent meals to meet their nutritional needs.

As pigs mature through the finishing phase, increases are typically seen in FIM, FR, and FRM, while the NVM, TNM, TM, and OTM show smaller changes or even decline [22,23,24,25]. Nonetheless, there is considerable variation in these trends across studies. For instance, Labroue et al. [22] noted a 28% rise in frequency of feeder visits for pigs between 40 and 60 kg, followed by an 11% decline as they grew to 90 kg, while Hyun et al. [23] and Gonyou and Lou [26] reported decreases in the number of meals and frequency of feeder visits of 17% and 24%, respectively, for pigs of similar weight ranges. On the other hand, Andretta et al. [24] and Carcò et al. [25] documented only modest fluctuations in the number of meals and frequency of feeder visits. The degree of increase in FIM differs between breeds (Figure 5) and research findings. For example, studies by Labroue et al. [22] and Andretta et al. [24] showed an approximate 60% boost in average daily feed intake as pigs grew from 30 to 100 kg and 35 to 95 kg in body weight, respectively. However, Carcò et al. [25] observed a more gradual increase in pigs between 47 and 145 kg, while Hyun et al. [23] found a 23% rise in average daily feed intake from 27 to 82 kg in body weight.

Regarding the daily feeding pattern, we observed that growing-finishing pigs showed two peaks of TNM throughout the day, one in the morning and one in the afternoon. This result agrees with findings by Hyun et al. [23] and Andretta et al. [24]. The preferred eating times were the periods from 8:00 h to 11:00 h and from 15:00 h to 17:00 h. However, the amount of feed consumed in a meal reached the highest values in the periods from 11:00 h to 14:00 h and 17:00 h to 21:00 h, whereas the FRM reached its maximum at 17:00 h. This result would indicate that animals eat less and more quietly when they visit the feeder more often.

### 4.2. Correlations Between Traits

Fernández et al. [27] suggested classifying growing-finishing pigs according to the number and size of the meals into “nibbler pigs” (i.e., those that have many short meals) or “meal eater pigs” (i.e., those that have few long meals) and according to the rhythm of ingesta into “fast” and “slow” eaters. The correlations between FB traits reported in the literature [22,23,27,28,29,30,31] and the results obtained in our study support the four feeding typologies (“nibbler-fast eater”, “nibbler-slow eater”, “meal-fast eater”, and meal-slow eater”), given the high and negative correlation between FR and the duration of the feeder visit (i.e., pig with a higher FR spend less time eating) and the low correlations between feeder visits per day and meal size with time spent eating per day and FR, suggesting no differences in FR between “nibbler” and “meal eater” pigs.

The correlation between growth and FB was negligible for most of the traits but FR, FRM, TNM, and FIM showed a correlation of 0.3–0.4 in magnitude. Therefore, fast eaters grow faster, reaching a higher final weight. This result partly agrees with findings by De Haer et al. [32], who reported that meal size and feeding rate significantly impact the growth performance of growing-finishing pigs, whereas the number of meals and the time spent eating have a poor influence. Those traits play a crucial role in the digestion and absorption of nutrients from feed [33,34]. Pigs that consume smaller meals at a slower pace tend to be leaner and exhibit a lower average daily gain. Additionally, Carcò et al. [25] found that feed intake and feeding rate were the FB parameters most strongly correlated with growth performance, showing a positive relationship with both average daily gain and final body weight.

The competition for feeding access is an important factor in FB and productive performance rather than group size. Individually housed pigs tend to eat their daily feed in smaller and more frequent meals. This behaviour leads to them spending more time eating due to a slower feeding rate compared to pigs housed in groups [32,35].

In our study, the only feeding behaviour traits that significantly influenced the feed efficiency were the FR and the FIM, showing a low and positive correlation with FCR and RFI. This suggests that pigs that eat more and faster tend to have a higher feed conversion rate. In accordance with our results, Andretta et al. [24] observed a negative correlation between meal size and feeding rate with the gain-to-feed ratio, indicating that both factors may impair nutrient utilization, likely due to their impact on passage rate or digestive enzyme activity [32,33]. Other studies also find weak correlations between meal size, feeding rate, and feed efficiency [22,23,25,31]. Le Naou et al. [36] discovered that pigs weighing 30 kg and fed the same amount twice daily in individual cages increased their average daily gain by 6.4% and improved their feed conversion ratio by 4% compared to pigs fed 12 times per day, supporting findings by Liu et al. [37]. This improvement could be attributed to the reduced maintenance energy requirements in pigs fed fewer meals per day [38]. Pigs fed once or twice a day tend to be less reactive to the excitement of feed distribution compared to those receiving several small meals, leading to less energy waste [39]. However, Schneider et al. [40], in a study on restricted feeding frequency, compared pigs fed either six or two meals per day (68 and 114 kg body weight pigs housed in pens of 10) and found that a higher number of meals positively affected performance, resulting in an increased average daily gain and improved feed conversion ratio.

Individuals with high FIM and IBM and low TNM are the ones that do not have priority access to feed and visit the feeder less times in comparison with other group mates. Therefore, they might be considered as subordinate pigs. So, it could be said that a dominant individual is a nibbler-slow eater pig, whereas a subordinate individual is a meal-fast eater pig. This result is consistent with findings by De Haer and Vries [33] regarding the FB of individually housed pigs relative to that of group-housed pigs. In addition, despite the fact that correlations are not high (0.2–0.3), although statistically significant, they indicate that the animals that eat more, occupy the feeder longer, and eat more times than their pen mates, are also the ones that eat mainly in the daytime period (i.e., between 8:00 h and 20:00 h), which is the preferred period, according to the feeding behaviour of individually housed animals [28]. These animals could be considered dominant, while subordinate animals would have to adapt their feeding schedule to the remaining time. This same social dynamic has been observed in dairy cattle by DeVries [41].

Hoy et al. [42] investigated the relationship between feeding behaviour and the social rank of animals within the group and thereby identified the feeding behaviour pattern of dominant/submissive animals. Contrary to our results, they concluded that the highest SR pigs took less visits to the feeder, stayed longer in it, and had higher FI per visit than the lowest SR pigs. However, in their study, the definition of dominant/subordinate animal already implies a certain pattern of feeding behaviour. However, Leiber-Schotte [43], cited by Hoy et al. [42], found that high ranking boars had more frequent and shorter visits with less feed intake than the lower ranking group mates, which is in agreement with our results. In addition, using individual records for the first time in group-housed rabbits [16], the same findings as the ones obtained in the present study were obtained: the most productive and efficient rabbits ate fewer times, consumed less, and spent less time in the feeder. Those animals also seemed to be subordinates, since they did not have priority access to feed and had the smallest share of resources [16]. On the contrary, rabbits that ate more, made more visits to the feeder, and spent more time in it to complete a meal were the ones that made their group mates more efficient and this was found to be heritable [44]. It is of interest for further research to explore the genetic basis of social ranking traits in pigs to elucidate if these traits may also respond to selection.

## 5. Conclusions

The results obtained in our study support the four feeding typologies previously described in the literature: “nibbler-fast eater”, “nibbler-slow eater”, “meal-fast eater” and meal-slow eater”. We found that pigs that eat more and faster tend to have a higher feed conversion rate, RFI, and higher final weight, whereas pigs that consume smaller meals at a slower pace tend to be leaner and exhibit a lower average daily gain. These findings can have important practical applications, as they can enable the easier and faster identification of the animals that are more efficient. In addition, animals that eat more and more times and occupy the feeder longer than their pen mates are also the ones that eat mainly in the preferred period. These animals could be considered dominant, while subordinate animals would have to adapt their feeding schedules to off-peak times. In practical terms, this finding can be used to optimize group housing strategies, improving animal welfare, reducing stress, and ensuring better performance. The findings of this study provide valuable insights into how feeding behaviours relate to growth and feed efficiency performance and social dynamics in pig populations.

## Figures and Tables

**Table 1:** Abbreviations and descriptions of feeding behaviour traits are used or calculated. When applicable, the formula for each calculation is provided.

	Abbreviation	Definition	Formula	Units
	ND	Number of days in the observation period		d
Feeding behaviouxviour	TNM	Number of meals		units
	NVM	Number of visits to the feeder to complete a meal		units
	TM	Time to complete a meal		s
	OTM	Occupation time in a meal		s
	FIM	Consumption per meal		g
	FR	Feeding rate	$\mathrm{FR}=\frac{\mathrm{FIM}}{\mathrm{OTM}}$	g/s
	FRM	Feeding rate to complete a meal	$\mathrm{FRM}=\frac{\mathrm{FIM}}{\mathrm{TM}}$	g/s
	IBM	Interval between meals		s
	TFI	Total feed intake in the observation period		g
Social Ranking	Position	The mean for all feeding events of the averages of the position in which each animal enters the feeder in each visit within a feeding event		units
	ratePrefTime.a	Ratio of visits to the feeder performed during the period from 8:00 to 20:00 h		Parts per unit
	ratePprefTime.b	Ratio of visits performed during the period from 7:00 to 11:00 and from 14:00 to 18:00 h		Parts per unit
	rateNV	The average of the ratio of the number of visits of the animal (NV individual) relative to the total number of visits in the pen (NV pen) computed on a daily basis	$\mathrm{rateNV}=\frac{\sum_{i}^{\mathrm{ND}}\frac{\mathrm{NV}\;\mathrm{individual}}{\mathrm{NV}\;\mathrm{pen}}}{\mathrm{ND}}$	Parts per unit
	rateNM	The average of the ratio of the number of meals of the animal (NM individual) relative to the total number of meals in the pen (NM pen) computed on a daily basis	$\mathrm{rateNM}=\frac{\sum_{i}^{\mathrm{ND}}\frac{\mathrm{NM}\;\mathrm{individual}}{\mathrm{NM}\;\mathrm{pen}}}{\mathrm{ND}}$	Parts per unit
	rateFI	The average of the ratio of the feed intake of the animal (FI individual) relative to the total feed intake in the pen (FI pen) computed on a daily basis	$\mathrm{rateFI}=\frac{\sum_{i}^{\mathrm{ND}}\frac{\mathrm{FI}\;\mathrm{individual}}{\mathrm{FI}\;\mathrm{pen}}}{\mathrm{ND}}$	Parts per unit
	rateOT	The average of the ratio of the time spent in the feeder by an animal (OT individual) relative to the total time spent in the feeder by all pen mates (OT pen) computed on a daily basis	$\mathrm{rateOT}=\frac{\sum_{i}^{\mathrm{ND}}\frac{\mathrm{OT}\;\mathrm{individual}}{\mathrm{OT}\;\mathrm{pen}}}{\mathrm{ND}}$	Parts per unit
Growth and meat quality	B	Body weight at the start of the follow-up period (~63 d)		g
	SW	Body weight at slaughter age (~163 d)		g
	BWG	Body weight gain in the monitoring period	BWG=SW−BWs	g
	ADGreg	Average daily gain as the slope of a linear model of body weight on age	lm() function of the R package stats	g/d
	MW	Metabolic weight	$\mathrm{MW}={\left(\frac{\mathrm{BWs}+\mathrm{SW}}{2}\right)}^{0.75}$	g
	Ham%	Percentage of ham in the carcass	Ham% = 100 × ham weight/weight of half carcass	%
	Fat%	Percentage of fat in the carcass	Fat% = 100 × back fat/weight of half carcass	%
	Loin%	Percentage of loin in the carcass	Loin% = 100 × loin weight/weight of half carcas	%
	Lean%	Percentage of lean in the carcass	Lean% = 25.08 + 0.73 × Ham% + 0.87 × Loin − 1.23 × Fat% [17]	%
Feed efficiency	FCR	Feed conversion ratio	$\mathrm{FCR}=\frac{\mathrm{TFI}}{\mathrm{BWG}}$	units
	RFI	Residual feed intake		g
	RG	Residual gain		g
	RIG	Residual intake and gain	RIG = RG − RFI	g

**Table 2:** Descriptive statistics of growth and feed efficiency traits for the three different pig breeds.

^1^ Trait (Units)	Breed 1		Breed 2		Breed 3	
	Mean	SD	Mean	SD	Mean	SD
ADGreg (g/d)	907.9	91.3	944.2	85.5	953.2	101.4
FCR (g/g)	2.47	0.22	2.44	0.20	2.43	0.23
RFI (Kg/d)	0	138.9	0	117.0	0	168.0

^1^ ADGreg: average daily gain regression; FCR: feed conversion ratio; RFI: residual feed intake.

## Data Availability

The data supporting this study are available from the authors upon reasonable request.

## Supplementary Materials

The following supporting information can be downloaded at: https://www.mdpi.com/article/10.3390/vetsci12020168/s1, Figure S1: Phenotypic correlations between productive performance (i.e., growth and feed efficiency), feeding, and social behaviour traits in pigs from breed 1, breed 2 and breed 3.
