# Supplementary material for: Feeding Behaviour in Group-Housed Growing-Finishing Pigs and Its Relationship with Growth and Feed Efficiency
Source: Vet Sci. 2025 Feb 13;12(2):168. doi: 10.3390/vetsci12020168 (PMC11860942; doi:10.3390/vetsci12020168)

Supplementary File S1

Feeding behaviour in group-housed growing-finishing pigs and its relationship with growth and feed efficiency

Correlation plots between productive performance (i.e. growth and feed efficiency), feeding and social behaviour traits in pigs from Breed 1, Breed 2 and Breed 3. Black crosses (X) indicate non-significant correlations between variables.

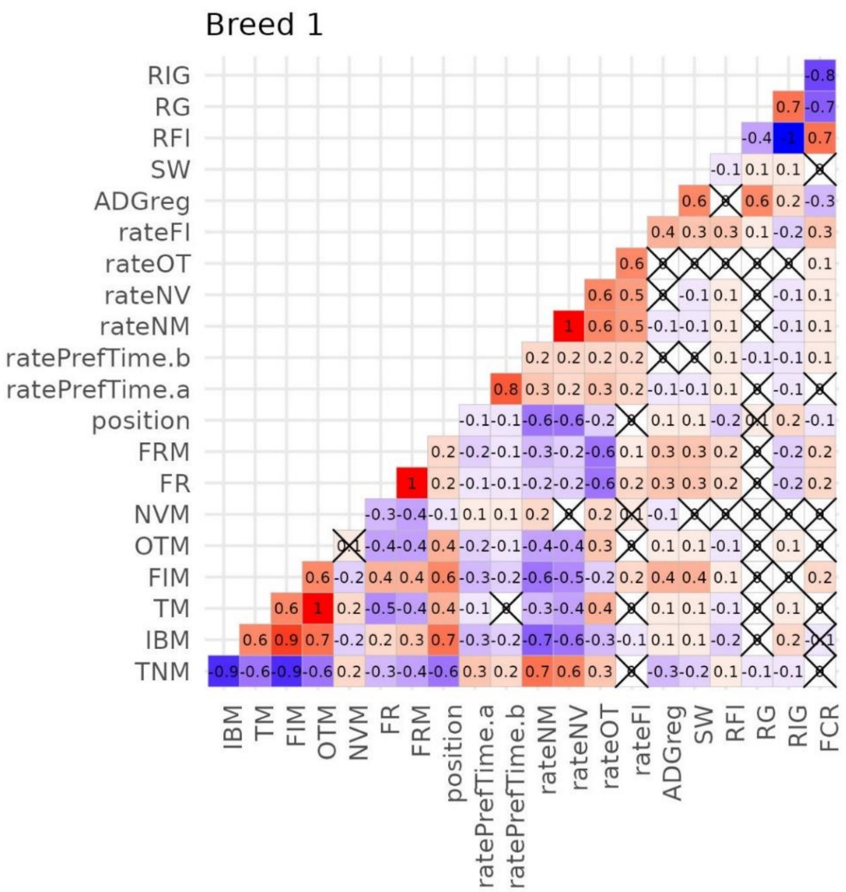

Breed 2

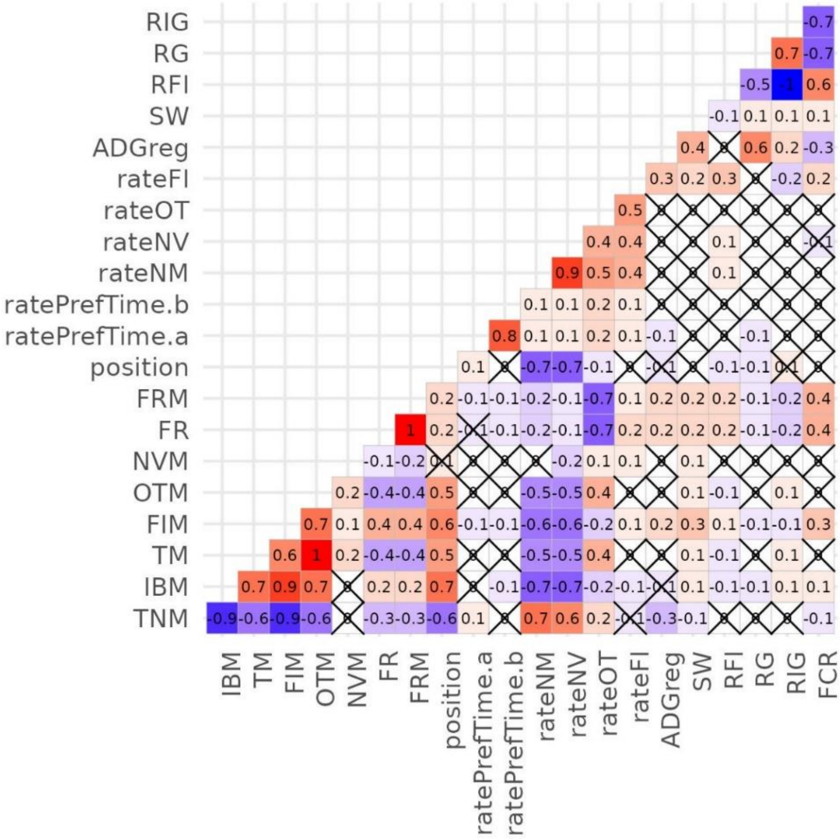

Breed 3

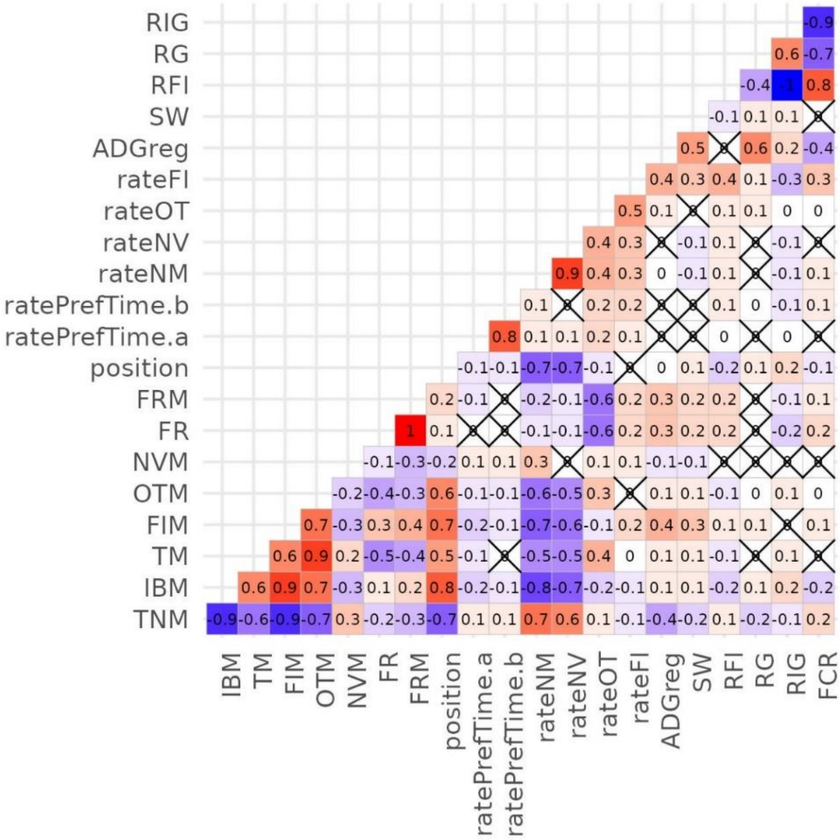

Supplement: Supplementary file 1 [file vetsci-12-00168-s001.zip › vetsci-3444397-supplementary.pdf]
